# Host protein glycosylation in nucleic acid vaccines as a potential hurdle in vaccine design for nonviral pathogens

**DOI:** 10.1073/pnas.1916131117

**Published:** 2020-01-06

**Authors:** Ahmet Ozdilek, Amy V. Paschall, Michelle Dookwah, Michael Tiemeyer, Fikri Y. Avci

**Affiliations:** ^a^Department of Biochemistry and Molecular Biology, University of Georgia, Athens, GA 30602;; ^b^Center for Molecular Medicine, University of Georgia, Athens, GA 30602;; ^c^Complex Carbohydrate Research Center, University of Georgia, Athens, GA 30602

**Keywords:** nucleic acid vaccines, protein glycosylation, infectious diseases

## Abstract

Nucleic acid vaccines introduce the genetic materials encoding antigenic proteins into host cells. If these proteins are directed into the secretory pathway with a signal/leader sequence, they will be exposed to the host’s glycosylation machinery, and, if their amino acid sequences contain consensus sequons for N-linked glycosylation, they may become glycosylated. The presence of host glycans on the proteins of microbial origin may prevent a strong protective immune response either through hindering access to key epitopes by lymphocytes or through altering immune responses by binding to immunoregulatory glycan-binding receptors on immune cells. Ag85A expressed by *Mycobacterium tuberculosis* (*Mtb*) is a bacterial surface protein that is commonly used in nucleic acid vaccines in multiple clinical trials. Here we show that, when Ag85A is expressed in mammalian cells, it is glycosylated, does not induce a strong humoral immune response in mice, and does not activate Ag85A-specific lymphocytes as highly as Ag85A natively expressed by the bacterium. Our study indicates that host glycosylation of the vaccine target can impede its antigenicity and immunogenicity. Glycosylation of the antigenic protein targets therefore must be carefully evaluated in designing nucleic acid vaccines.

DNA vaccines, recombinant vector vaccines, and messenger RNA vaccines comprise nucleic acid vaccines (1). This next-generation vaccine approach adopts a different modus operandi than other vaccine types in use today: Rather than using whole pathogens or purified protein products, these vaccines introduce the genetic materials encoding antigenic, pathogen-associated proteins into the host cells. Unlike viral proteins, proteins of nonviral pathogens are not expressed in host cells upon infection. We postulated that, when nucleic acid vaccines are used for nonviral pathogens, the proteins expressed by host cells would be structurally and immunogenically different from native pathogen protein, due to the elaboration of host posttranslational modifications (PTMs), especially glycosylation for targets that enter the secretory pathway. Glycans may hinder or alter important epitopes and/or down-regulate immune responses through many regulatory immune cell receptors (2). Therefore, the glycans on the proteins expressed through nucleic acid vaccines may undesirably influence the immune responses against the antigen. Here, we investigated the impact of host glycosylation on the immunological properties of *Mtb* surface protein, Ag85A, a target protein used in multiple clinical vaccine trials (3). In three different clinical trials, two viral vector-based Ag85A vaccine candidates were not protective or immunogenic among the participants (4–6). Toward understanding the results of these trials, we show that Ag85A expressed in mammalian cells is glycosylated and immunization with this recombinant Ag85A generates a significantly reduced immune response compared to immunizations with its native form expressed in *Mtb*.

## Results

**Fig. 1.**
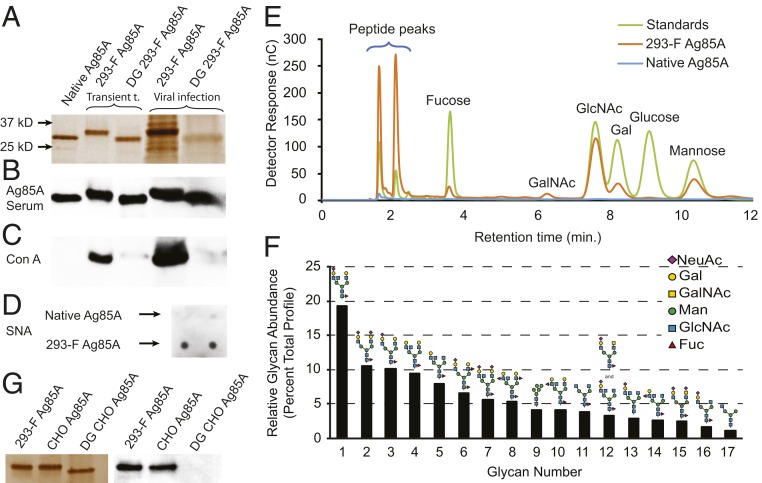
Ag85A is glycosylated when expressed in mammalian cells. (*A*) Sodium dodecyl sulfate polyacrylamide gel electrophoresis (SDS/PAGE) analysis of purified native/recombinant Ag85A molecules with or without PNGase F treatment. (*B*) Western blot of proteins in *A* probed with sera from mice immunized with purified 293-F Ag85A. (*C*) Lectin blot of proteins in *A* probed with ConA. (*D*) Dot blot of native Ag85A and 293-F Ag85A with SNA. (*E*) Monosaccharide analysis of native Ag85A and purified 293-F Ag85A glycans by acid hydrolysis followed by HPAEC-PAD. (*F*) N-glycan analysis of 293-F Ag85A. N-glycans were released enzymatically, permethylated, and analyzed by NSI-MSn. The relative abundances of all individual glycans detected at quantifiable levels are presented as the percent of the total glycan profile contributed by each indicated glycan. (*G*) SDS/PAGE analysis and lectin blot (ConA) of 293-F Ag85A, CHO Ag85A, and deglycosylated CHO Ag85A.

To investigate the effects of mammalian/host glycosylation on immune responses, we expressed Ag85A in human embryonic kidney cells (HEK 293-F) through either transient transfection or adenovirus 5 infection (designated as 293-F Ag85A). The 293-F Ag85As treated with PNGase F, an enzyme that removes N-glycans on proteins expressed in vertebrate cells, showed a molecular weight shift (Fig. 1*A*) indicating that 293-F Ag85A is N-glycosylated. To confirm that all of the major bands displayed in Fig. 1*A* are Ag85A, Western blotting was performed, probing with serum from mice immunized with purified 293-F Ag85A (Fig. 1*B*). In a lectin blotting experiment, a mannose binding lectin, Con A (ConA), reacted with 293-F Ag85A but not native Ag85A (Fig. 1*C*). Upon deglycosylation, 293-F Ag85A binding is ablated (Fig. 1*C*). As it is often indicated in immunoregulation (2, 7, 8), we next confirmed the presence of terminal sialic acid on 293-F Ag85A (expressed via transient transfection) in a dot blot by probing with Sambucus nigra lectin (SNA), a lectin that binds to α2,6-linked sialic acids (Fig. 1*D*). Monosaccharide composition of glycans of native and 293-F Ag85A were then analyzed by high-performance anion exchange chromatography with pulsed amperometric detection (HPAEC-PAD), which revealed the presence of GalNAc, GlcNAc, mannose, galactose, and fucose as the neutral monosaccharides on the 293-F Ag85A, while no monosaccharide is detected on native Ag85A (Fig. 1*E*). To acquire a higher-resolution understanding of 293-F Ag85A glycosylation, its N-glycans were released by PNGaseF digestion and analyzed by multidimensional mass spectrometry (NSI-MSn). Among the detected glycans, 15 of 17 were complex type, variably terminated with sialic acid (NeuAc) (Fig. 1*F*). To confirm its glycosylation in an alternate mammalian expression system, we expressed Ag85A in Chinese Hamster Ovarian (CHO) cells and analyzed by lectin blotting (Fig. 1*G*).

**Fig. 2.**
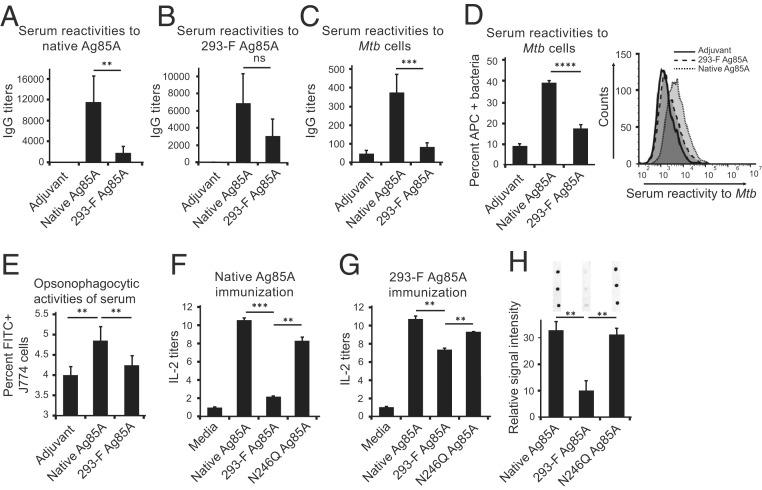
The 293-F Ag85A elicits weaker immune responses than native Ag85A. Serum IgG reactivities of mice immunized with adjuvant alone, native Ag85A, or 293-F Ag85A against (*A*) native Ag85A, (*B*) 293-F Ag85A, or (*C*) gamma irradiated *Mtb*, determined by ELISA. Serum IgG titers were reported as the reciprocal dilution that results in an optical density of 0.5 at 450 nm. (*D*) Intact *Mtb* binding of immune sera analyzed by flow cytometry. Overlaid histograms represent data from four technical repeats. (*E*) Flow cytometry analysis for opsonophagocytic activities of immune sera coincubated with FITC-labeled *Mtb* and J774 mouse macrophages. Splenic mononuclear cells harvested from mice immunized with native Ag85A (*F*) or 293-F Ag85A (*G*) were assessed for their IL-2 production upon stimulation with native, 293-F, or N246Q Ag85A by ELISA. IL-2 concentrations were normalized to media. (*H*) Serum IgG reactivities of mice immunized with native Ag85A against native, 293-F, or N246Q Ag85A were determined in a dot blot analysis, and dots were quantified with ImageJ software. Data shown are from the same membrane with the same exposure time. Statistical significance was determined with the two-tailed Student’s *t* test. ****P < 0.0001; ***P < 0.001; **P < 0.01; ns, not significant.

To investigate the differences in antigenicity and immunogenicity of native and 293-F Ag85A, three groups of mice were immunized with adjuvant alone (250 µg of dimethyldioctadecylammonium bromide per dose), native Ag85A, or 293-F Ag85A (10 µg per dose). A week after the booster immunization, sera were collected, and antibody reactivities against native or 293-F Ag85A were tested by enzyme-linked immunosorbent assay (ELISA). Immunization of mice with native Ag85A yielded significantly higher IgG titers than sera from 293-F Ag85A-immunized mice when plates were coated with native Ag85A (Fig. 2*A*) or 293-F Ag85A (Fig. 2*B*). We next tested the serum IgGs for their binding to bacterial cells, in a whole-cell ELISA. Sera obtained from native Ag85A-immunized mice showed significantly higher IgG binding to plates coated with gamma-irradiated *Mtb* than sera from 293-F Ag85A immunized mice (Fig. 2*C*). The differential binding of serum antibodies to bacteria observed in ELISA was also confirmed by flow cytometry (Fig. 2*D*). These results demonstrate that immunization with 293-F Ag85A does not evoke a humoral immune response to bacteria as strongly as immunization with native Ag85A does. One of the effector functions of antigen-specific antibodies is to enhance phagocytosis and clearance of pathogens. Therefore, we compared the function of antibodies raised against native or 293-F Ag85A in an opsonophagocytosis assay. For this assay, we incubated fluorescein isothiocyanate (FITC)-labeled *Mtb* with sera from different immunization groups. Opsonized bacteria were then incubated with J774 mouse macrophages. Flow cytometry was used to quantify FITC^+^ J774 cells, indicative of phagocytosed bacteria. In parallel to the sera reactivities against whole bacteria, serum from native Ag85A immunization induced a significantly higher degree of binding/uptake of bacteria compared to serum from 293-F Ag85A immunization (Fig. 2*E*). To test Ag85A-specific interleukin-2 (IL-2) production as a measure of lymphocyte activation (9), splenic mononuclear cells harvested from mice immunized with native Ag85A or 293-F Ag85A were cultured in the presence of native or 293-F Ag85A (10 µg antigen per mL), and, after 3 days, the IL-2 levels in the culture media were quantified. Native Ag85A stimulation induced significantly higher IL-2 secretion than 293-F Ag85A stimulation, regardless of which Ag85A variant was used in the immunization (Fig. 2 *F* and *G*). To assess the recovery of T cell stimulation by the removal of N-linked glycan, we expressed Ag85A in HEK 293-F cells in its nonglycosylated form after mutagenesis of the N-glycosylation site (N246Q Ag85A). As demonstrated in Fig. 2 *F* and *G*, N246Q Ag85A stimulates higher IL-2 responses compared to 293-F Ag85A. We also tested serum IgGs from native Ag85A-immunized mice for their binding to N246Q Ag85A (60 ng antigen per dot) and showed that native Ag85A serum reacts with N246Q Ag85A significantly more than 293-F Ag85A (Fig. 2*H*).

## Discussion

Since their introduction, nucleic acid vaccines have shown great promise and have been quickly propelled to clinical trials (10). The utilization of a host’s protein biosynthesis machinery through introduction of the antigenic gene potentially provides a variety of advantages over traditional subunit vaccines, including vaccine cost-effectiveness, stability, and persistence of immunogenicity (1, 3, 11). However, relying on a host’s cellular machinery to produce the antigens comes with a potential caveat: Mammalian posttranslational machinery—namely glycosylation—may decorate the antigenic protein with self-glycans. This may, in turn, yield undesirable antigens for immune recognition and poor immunogens for eliciting protective immune responses. Moreover, decoration of antigenic protein with host glycans may trigger suppressive immune responses, since host glycans are known for their immunoregulatory properties (2, 7, 8). The down-regulatory effects of host-associated glycans are also exploited by many pathogens, which display these structures on their surfaces to mimic “self” and thus evade immune recognition (12–16).

Through the use of a clinical nucleic acid vaccine candidate, Ag85A, this study demonstrates that, when nucleic acid vaccines are used to express a bacterial protein in mammalian host cells, the protein product can be glycosylated by host glycosylation machinery (Fig. 1). In turn, these glycoprotein antigens may elicit impaired and/or tolerogenic immune responses, due to their dampened antigenicity and immunogenicity or their newly acquired immunosuppressive properties (Fig. 2 *A*–*E*). Thus, glycosylation is a critical PTM to consider in designing vaccine strategies. An ideal vaccine target has to share the same antigenic determinants as the native antigen expressed on the surface of the pathogen. Removal of N-linked sequons is potentially a reasonable strategy toward avoiding this complication, as the nonglycosylated N246Q Ag85A displays immunological properties comparable to native Ag85A (Fig. 2 *F*–*H*). It is also critical to evaluate the role of mammalian glycosylation in altering cellular immunity. Previously, a nucleic acid vaccine employing Ag85A DNA induced CD8+ T cell responses (17). In light of these findings, it is imperative to investigate the potential impact of mammalian glycosylation on cell-mediated immune responses. Moving vaccine candidates from preclinical investigation to clinical phase studies comes with substantial cost and effort. We believe the findings of this study serve as essential criteria for the design of future nucleic acid vaccines and projecting their ultimate success of protectivity.

## Materials and Methods

Native Ag85A and gamma-irradiated *Mtb* were obtained from Biodefense and Emerging Infections Research Resources Repository. For HEK 293-F expressions of Ag85A, we used tissue plasminogen activator leader sequence to direct Ag85A into the secretory pathway. To generate adenoviruses for the expression of Ag85A, we utilized AdEasy system (18). After being expressed by mammalian cells, 293-F Ag85A, CHO Ag85A, and N246Q Ag85A−GFP (green fluorescent protein) were purified using nickel-nitrilotriacetic acid (Ni−NTA) affinity columns. N246Q Ag85A was cleaved from GFP by tobacco etch virus (TEV) protease and purified. Antibodies are as follows: goat anti-mouse IgG, BioLegend, Cat# 405301; anti-IL-2 capture antibodies, BioLegend, Cat# 503704; biotinylated anti-IL-2 detection antibodies, BioLegend, Cat# 503804; and avidin-HRP, BioLegend, Cat#405103. Flow cytometry antibodies are as follows: Alexa Fluor 647 goat anti-mouse IgG antibodies, BioLegend, Cat# 405322. Experiments involving vertebrate animals were performed under an Institutional Animal Care and Use Committee approved animal use protocol. University of Georgia is fully accredited by the American Association for Accreditation of Lab Animal Care (AAALAC). Veterinary care for all research animals is under the supervision of the Campus Office of Veterinary Services in accordance with US Department of Agriculture, Public Health Service and AAALAC requirements.

### Data Availability.

All relevant data are included herein.
